# Supplementary material for: Evaluating Knowledge of Human Microbiota among University Students in Jordan, an Online Cross-Sectional Survey
Source: Int J Environ Res Public Health. 2021 Dec 17;18(24):13324. doi: 10.3390/ijerph182413324 (PMC8708365; doi:10.3390/ijerph182413324)

Bacteria in health and disease questionnaire

402	06:38	Closed
Responses	Average time to complete	Status

1. Age العمر

402
Responses

Latest Responses
"19"
"20"
"21"

2. Sex الجنس

Insights

Male	155
Female	247

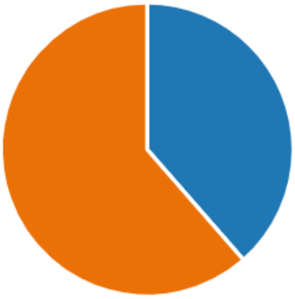

3. Which School category do you belong to ? إلى أي فئة من كليات الجامعة تنتمي كليتك

Insights

Health schools (e.g. Medicine, ...	284
Scientific schools (e.g. Science,...	94
Humanities schools (e.g. Arts, ...	24

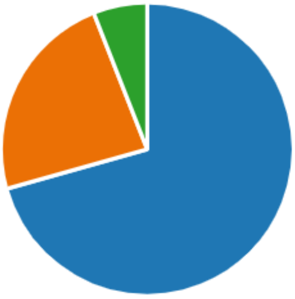

4. Are you a medical student?

Insights

- Yes 237
- No 47

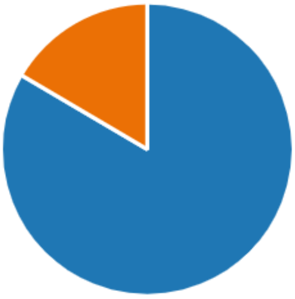

5. Year of study السنة الدراسية

Insights

- First year 97
- Second year 206
- Third year 73
- Fourth year 18
- Fifth year 4
- Sixth year 4

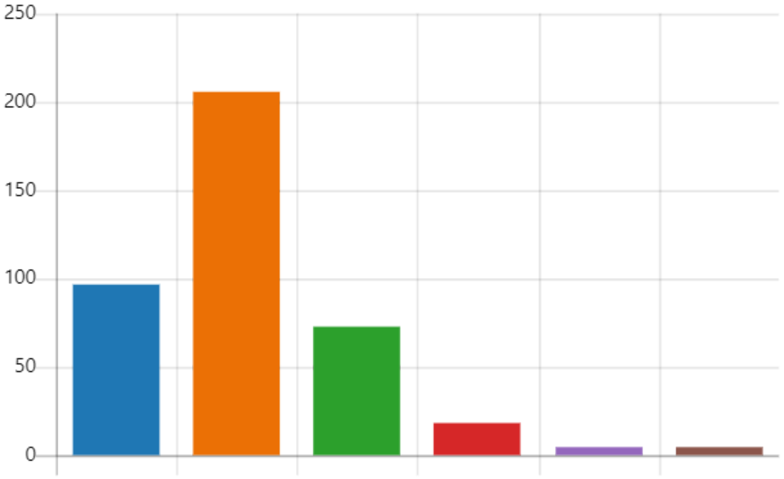

6. How do you rate your knowledge about bacteria? كيف تقيم معرفتك ب البكتيريا؟

- Poor knowledge, if you never ... 43
- Basic knowledge, from person... 202
- Advanced knowledge, if you t... 157

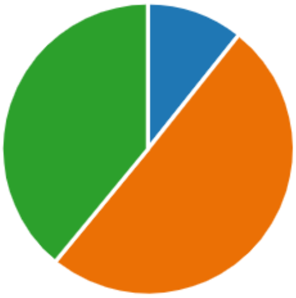

7. How much do you agree with the following statements? إلى أي مدى توافق على العبارات التالية

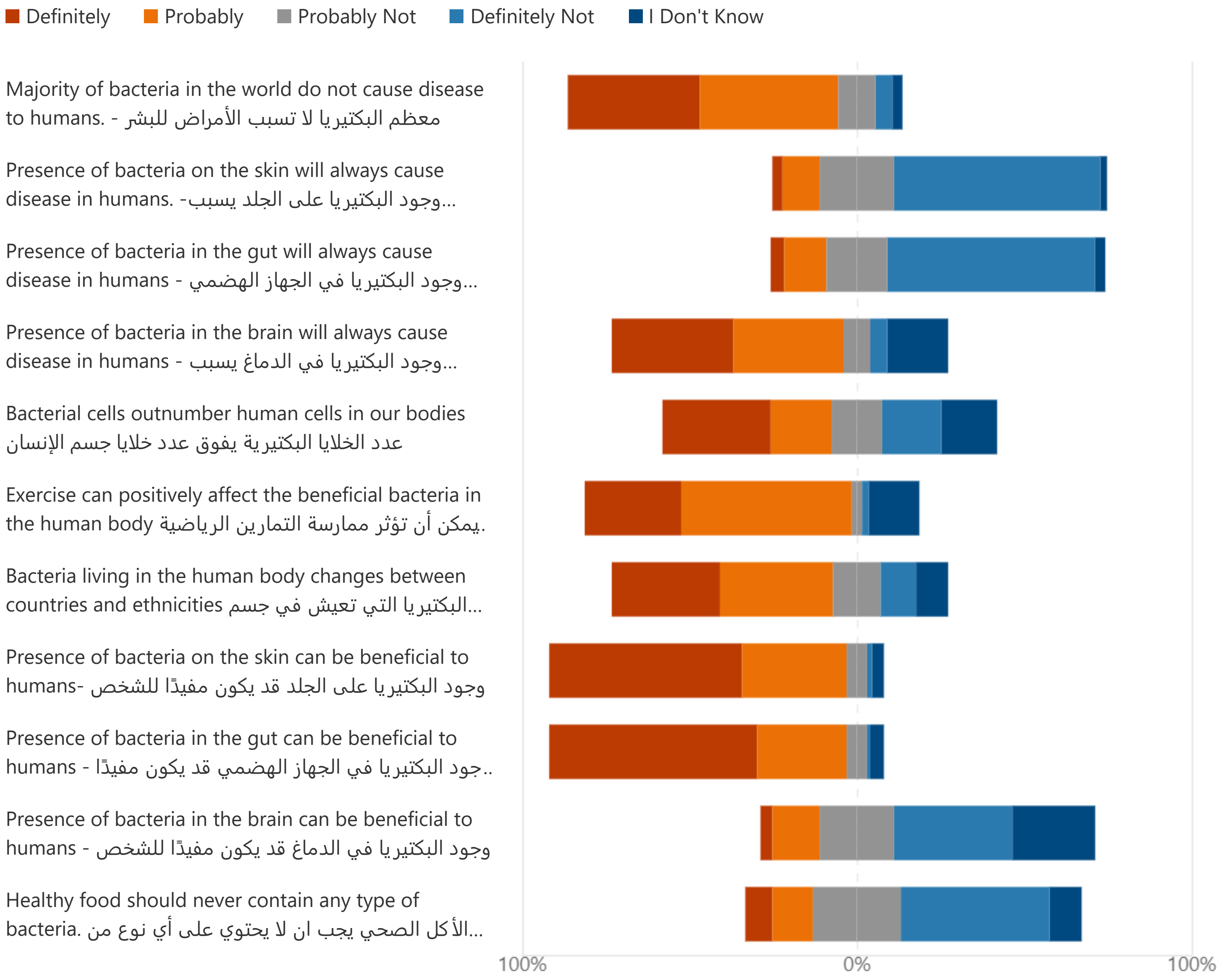

8. How much do you agree with the following statements? إلى أي مدى توافق على العبارات التالية؟

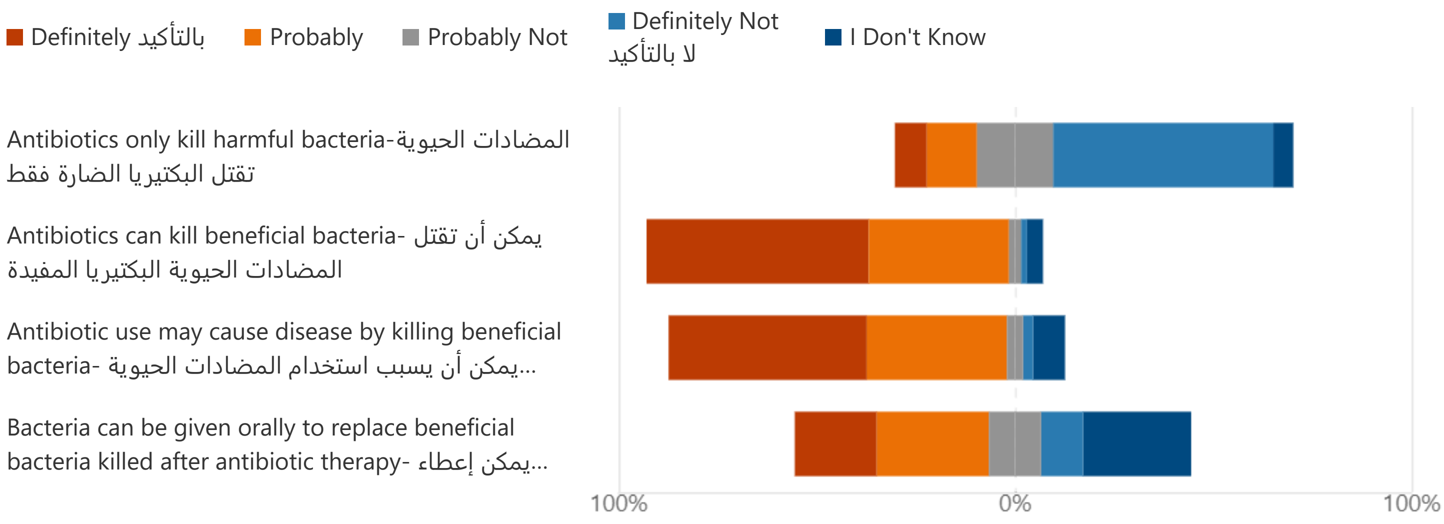

9. How much do you agree with the following statements? إلى أي مدى توافق على العبارات التالية؟

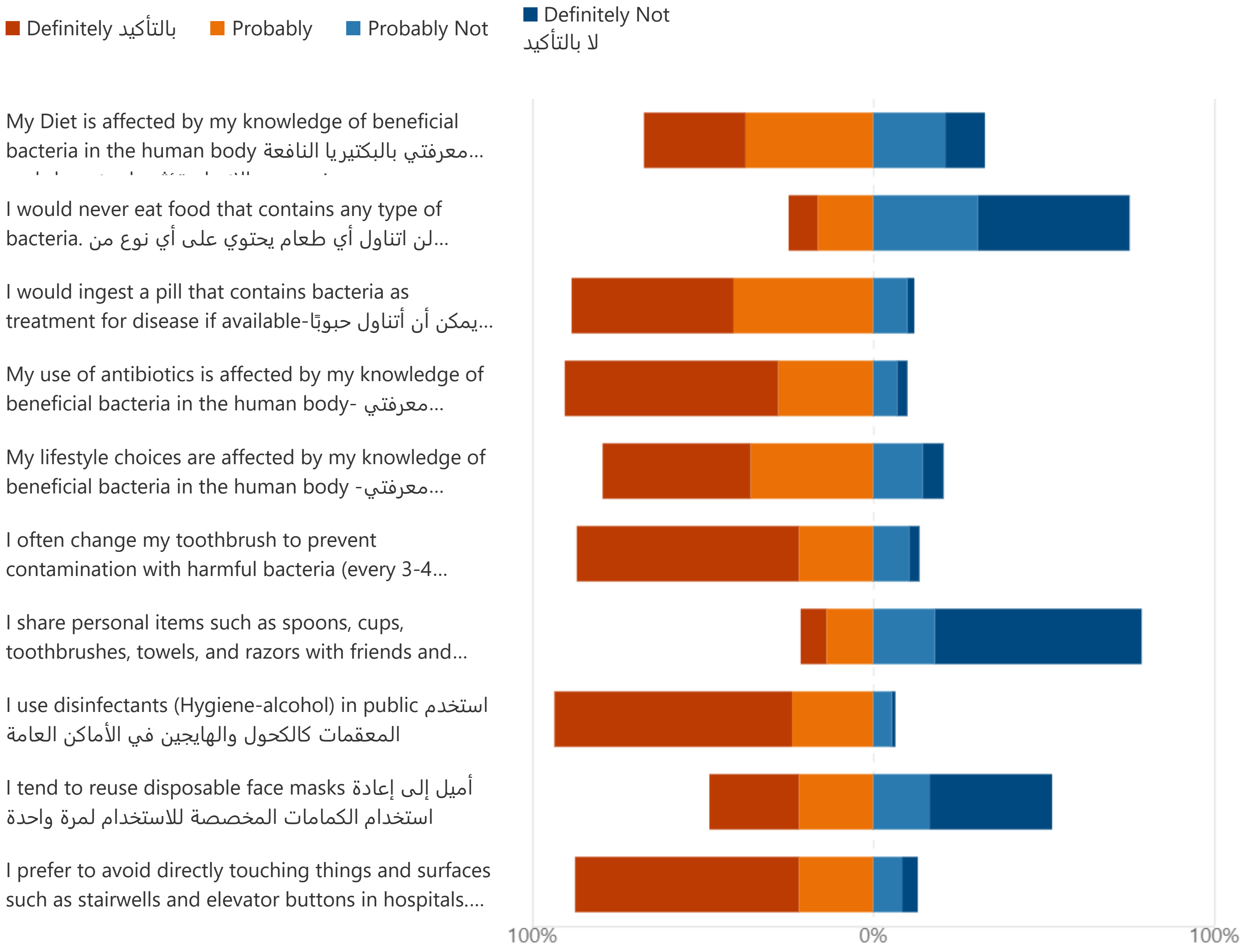

10. I would like to learn more about how bacteria in the human body can affect health and disease- سأكون مهتمًا بمعرفة المزيد عن البكتيريا النافعة في جسم الإنسان، وكيف يمكن أن تؤثر على الصحة والمرض

Yes	375
No	27

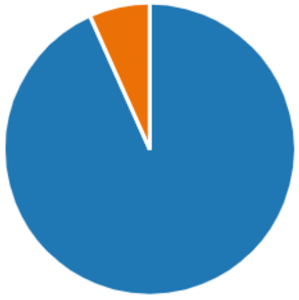

11. If yes, which of the following sources would you use? (You can choose multiple options) - إذا كانت إجاباتك نعم، ما هي الوسيلة التي تفضلها لذلك؟ (يمكن اختيار اختيارات متعددة)

Social media- تواصل الاجتماعي..	183
Healthcare workers- اية الصحية...	149
News outlets- مصادر الأخبار	61
Trusted medical sources (e.g. ...	284
Other	8

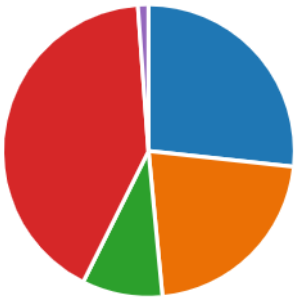

Supplement: Supplementary file 1 [file ijerph-18-13324-s001.zip › ijerph-1453715-supplementary.pdf]
